# The Neonatal Screening Program in Brazil, Focus on Sickle Cell Disease (SCD)

**DOI:** 10.3390/ijns5010011

**Published:** 2019-01-26

**Authors:** Ana C. Silva-Pinto, Maria Cândida Alencar de Queiroz, Paula Juliana Antoniazzo Zamaro, Miranete Arruda, Helena Pimentel dos Santos

**Affiliations:** 1 Policy of Integral Attention to People with Sickle Cell Disease (PIAPSCD), Technical Advisory Council for Sickle Cell Disease (TAC-SCD), CGSH/DAET/SAS, Ministry of Health, Asa Norte Brasília 70719-040, Brazil; 2 Regional Blood Center of Ribeirão Preto, HC-FMRP, University of Sao Paulo (USP), Campus Universitário, Ribeirão Preto 14049-900, Brazil; 3 National Newborn Screening Program Policy (NNSPP)/CGSH/SAS, Ministry of Health, Asa Norte Brasília 70719-040, Brazil; 4 State Health Secretariat, State of Pernambuco, Recife 50751-530, Brazil; 5 Newborn Screening Program, APAE-Salvador, Salvador 41830-141, Brazil

**Keywords:** neonatal screening, sickle cell disease, hemoglobinopathies

## Abstract

Since 2001, the Brazilian Ministry of Health has been coordinating a National Neonatal Screening Program (NNSP) that now covers all the 26 states and the Federal District of the Brazilian Republic and targets six diseases including sickle cell disease (SCD) and other hemoglobinopathies. In 2005, the program coverage reached 80% of the total live births. Since then, it has oscillated between 80% and 84% globally with disparities from one state to another (>95% in São Paulo State). The Ministry of Health has also published several Guidelines for clinical follow-up and treatment for the diseases comprised by the neonatal screening program. The main challenge was, and still is, to organize the public health network (SUS), from diagnosis and basic care to reference centers in order to provide comprehensive care for patients diagnosed by neonatal screening, especially for SCD patients. Considerable gains have already been achieved, including the implementation of a network within SUS and the addition of scientific and technological progress to treatment protocols. The goals for the care of SCD patients are the intensification of information provided to health care professionals and patients, measures to prevent complications, and care and health promotion, considering these patients in a global and integrated way, to reduce mortality and enhance their quality of life.

## 1. Background

The Brazilian population is currently estimated at over 208 million people. For the most part, three distinct peoples form the ethnic backgrounds of this population: the Native Americans (Indians), the Portuguese, and the Africans. Population data from the 2010 Demographic Census showed that 50.7% of the Brazilian population is made up of Afro-Americans, 47.7% Caucasians, and 0.7% Indians (0.9% unspecified). Since the population is broadly miscegenated, all national programs must be universalized and not directed at a specific portion of the population. Therefore, the neonatal screening program is national and universal, aiming to reach 100% of live births in the country [1].

## 2. The Neonatal Screening Program

The history of neonatal screening (NS) in Brazil goes back to 1976, when APAE-São Paulo began neonatal screening for phenylketonuria (PKU). Several pilot NS initiatives were then initiated independently without coordination nor standardization among Brazilian states. In 1992, the screening for PKU and congenital hypothyroidism (CH) was included in the public health system, known as SUS [2]. In 1998, the State of Minas Gerais was the first to introduce a universal State NS Program for sickle cell disease (SCD) in the already existing programs for PK and CH [3].

However, the recognition of NS as a specific public health program only happened in 2001 after the founding of the National Neonatal Screening Program (NNSP) coordinated by the Ministry of Health [4]. The program led to the definition of standards and protocols for the whole country, and placed screening as a global health action aimed at preventing child health problems not only through testing to detect the disease but also the active search of suspected cases, diagnostic confirmation, treatment, and follow-up of patients [5]. The NNSP initially focused on PKU, CH, cystic fibrosis, SCD, and other hemoglobinopathies. In 2014, the program was extended to screening for biotinidase deficiency and congenital adrenal hyperplasia.

The Ministry of Health defined priorities and divided the program into four phases, accrediting the states according to each one’s capacity. From 2001 onward, reference services were certified sequentially in one of the four phases of the NNSP. Table 1 shows the years at which each of the phases reached universal implementation in all the states and the Federal District.

**Figure 1:**
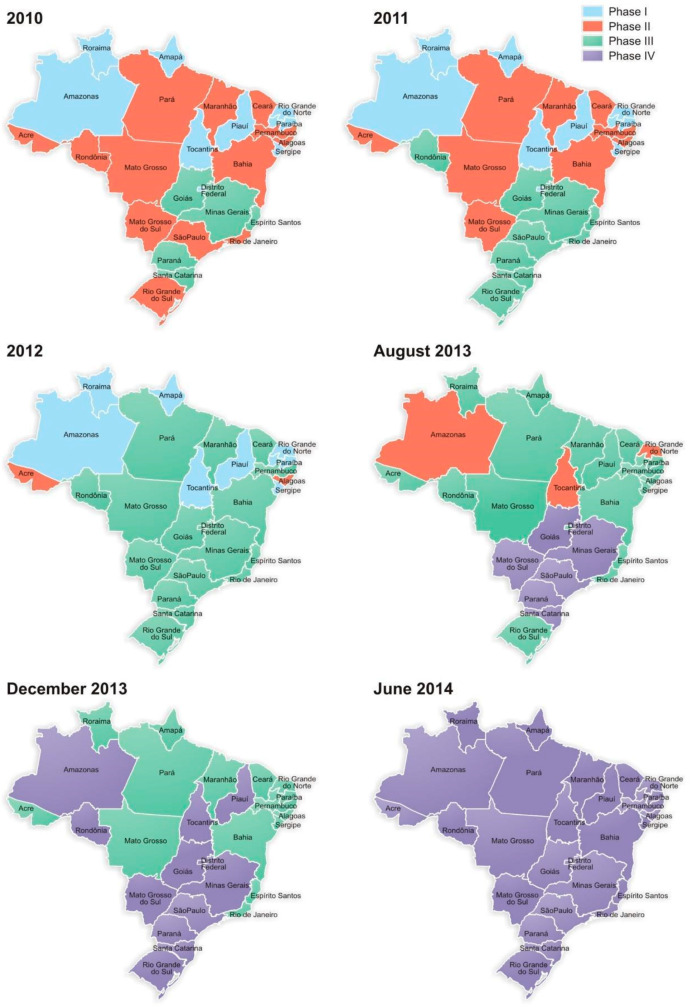
Expansion of the neonatal screening program in Brazil from 2010 to 2014 regarding the four phases of the National Neonatal Screening Program (NNSP).

Figure 1 illustrates the sequential expansion of the neonatal screening program in the various states of Brazil between 2010 and 2014.

**Figure 2:**
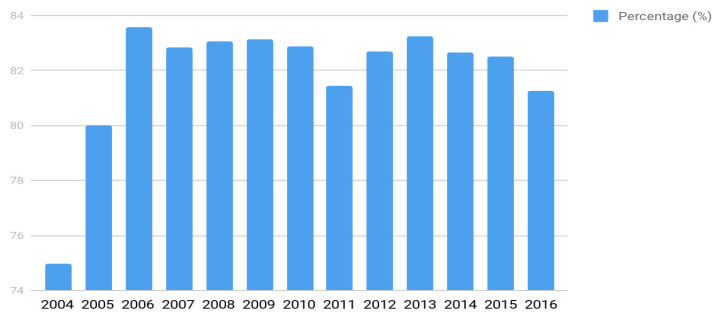
The annual rate of the program coverage from 2004 to 2016.

The coverage rate of the program (percentage of the screened live births) varied over the years (Figure 2). Since 2006, it has remained above 85% at the national level, with few variations. This figure relates to variations in the health care network capacity from state to state and to variations in the population’s access to it. In São Paulo State, for example, the percentage of coverage reaches over 95%.

## 3. Resolutions and Guidelines from the Brazilian Ministry of Health

In addition to the implementation of neonatal screening and with the goal of standardizing standards of care, the Ministry of Health has published several guidelines for clinical follow-up and treatment for each of the diseases included in the neonatal screening program (see, for example, the Clinical Protocol and Therapeutic Guidelines for Sickle Cell Disease [6]). These include the description of the general concepts for each disease, diagnostic criteria, inclusion and exclusion criteria, treatment and mechanisms of regulation, and control and treatment evaluation. Altogether, they establish a national policy and should be used by the state and municipal Health Secretariats for the regulation of care access, authorization, registration, and reimbursement of the corresponding procedures.

## 4. The Sickle Cell Disease Network of Care Inside SUS: From Neonatal Screening to Follow-Up in Reference Centers

Health is inscribed in the national Constitution as a right for everyone and a duty of the Brazilian Republic. This was the framework for the creation of the Unified Health System, known as SUS, which contemplates the guidelines for health promotion and provision of services and establishes the community participation and transfer of federal resources to other jurisdictions [7,8]. Despite the great barriers that SUS has faced since its conception, it has managed to establish itself as a system that proposes to be an institutional redistributive model, guaranteeing universal coverage. In this context, the inclusion of sickle cell disease (SCD) and other hemoglobinopathies in the National Neonatal Screening Program (NNSP) instituted by the SUS in 2001 shows recognition of this group of pathologies as a national public health issue [9].

The proportion of live births with SCD varies widely among the states, being more frequent in states with a higher concentration of African descendants. For example, in Bahia for every 650 births, 1 child has SCD, followed by Rio de Janeiro (1:1200) and then Pernambuco, Maranhão, and Minas Gerais, with 1:1400. Table 2 shows the incidence of newborns affected by SCD in 12 states representative of the four most populated regions of Brazil (the fifth region being the North that covers the Amazonian forest).

Building up the current network and system of care for the people with SCD in Brazil has been a long process, in some ways reminiscent of the 1970s struggle for civil rights and equality for access to care in the USA that led to the US *National Sickle Cell Anemia Control Act* in 1972 [10,11]. In the 1980s, Brazilian representative entities for the the defense of the rights of family and people with SCD took the first steps to formulate claims, initially at the local level, and then expanded their coordination with the formation in 2001 of the National Federation of Associations of People with Sickle Diseases (FENAFAL). The march Zumbi dos Palmares against Racism, for Citizenship and for Life, held in Brasilia in 1995, was an important milestone in the struggle of black people against the Brazilian State for affirmative action and against racism in terms of health care. From this action, important policies emerged, including the Policy of Integral Attention to People with Sickle Cell Disease (PIAPSCD) [12]. The major goals were to change the natural history of SCD in Brazil, by reducing morbidity and mortality, promoting greater survival, and improving the quality of life for people with SCD. Commitments were made to provide genetic guidance, safeguarding reproductive rights of people with the sickle cell trait, and to disseminate information about SCD to the general population.

In 2006, the Ministry of Health established a Technical Advisory Council for Sickle Cell Disease (TAC-SCD) to help states and municipalities organize their programs of care for people with SCD. TAC is composed of specialists, managers of state and municipal jurisdictions of SUS, and representatives of educational, research, and assistance institutions involved in the care of people with SCD who are invited to act voluntarily. There is also a representation of the users chosen by FENAFAL. TAC is responsible for producing the guidelines for the management of SCD and its complications, genetic guidelines, family guidelines, guidelines for oral health and on the role of nursing, which are printed and distributed in the reference centers and in the basic SUS network. These materials are available electronically in the SUS Virtual Library (VL). The members of TAC also conduct training courses, workshops, and symposia on SCD in all regions of the country, contributing to the continuing education of health professionals.

Within SUS, the standardized attention to people with SCD encompasses all levels from early diagnosis to bone marrow transplantation. Assistance begins in basic care with the early diagnosis free of charge through the NNSP that links the affected child to the basic health unit, responsible for the first procedures of prevention such as prophylaxis with penicillin initiated in the first month of life, immunobiological approaches, and then referral to the regional Specialized Care Service. In most states, the reference center is the Blood Center, which is normally integrated with the local health network to meet other health needs, such as the use of hydroxyurea, transcranial Doppler, and all procedures of greater complexity. The implementation of the SCD network in all the states is intended to guarantee decentralized care beginning with diagnosis, with the assistance of a multi-professional and multidisciplinary team, providing health education with a focus on self-management and access to specialized and high complexity care in all the 26 states and the Federal District [13].

## 5. Challenges for the Full Implementation of the SCD Care Network

Despite major advances, the organization of a robust health care network for people with SCD is still a major challenge to SUS. Only a network with the potential for the collective construction of solutions is able to cope with the complexity of demands and to guarantee the promotion of autonomy and citizenship to people with SCD. The disease has not yet reached the level of visibility pursued that is necessary to provide its target population with the right to health they deserve to extend their life horizon with personal and social well-being.

Notably, considerable gains have already been achieved, including the implementation of neonatal screening at the national level together with scientific and technological progress with respect to treatment. Preliminary data on the effect of hydroxyurea treatment and mortality rates have been published [14,15,16]. However, more precise epidemiological data covering the entire country are still needed. A national registry for SCD patients is being set up to collect more reliable information to evaluate the system: percentage of new cases that actually enter follow-up at reference centers, immunization rate, percentage of patients that started pneumococcal prophylaxis, rate of children screened with transcranial Doppler, use of hydroxyurea, and death rates at the country level. What is sought to bring attention to people with SCD is the intensification of information provided, measures to prevent injuries, and care and health promotion, considering these patients in a global and integrated way, to reduce mortality and enhance their quality of life.

## Figures and Tables

**Table 1:** The implementation of neonatal screening divided into phases, and its year of universalization.

Phases	Diseases	Year of National Implementation
Phase I	Phenylketonuria and congenital hypothyroidism	2006
Phase II	Sickle cell disease and other hemoglobinopathies	2013
Phase III	Cystic fibrosis	2013
Phase IV	Congenital adrenal hyperplasia and biotinidase deficiency	2014

**Table 2:** Incidence of sickle cell disease and sickle cell trait in some Brazilian states.

Regions of Brazil	State	Incidence/Live Births	Incidence of the S Mutation
Northeast	Maranhão	1:1400	1:23
	Pernambuco	1:1400	1:23
	Bahia	1:650	1:17
Center-west	Goiás	1:1400	1:28
	Mato Grosso do Sul	1:8300	1:70
Southeast	Minas Gerais	1:1400	1:30
	Espírito Santo	1:1800	1:28
	Rio de Janeiro	1:1200	1:21
	São Paulo	1:4000	1:35
South	Paraná	1:13,000	1:65
	Santa Catarina	1:13,000	1:65
	Rio Grande do Sul	1:13,500	1:65
